# How teacher and classmate support relate to students’ stress and academic achievement

**DOI:** 10.3389/fpsyg.2022.992497

**Published:** 2022-11-28

**Authors:** Frances Hoferichter, Stefan Kulakow, Diana Raufelder

**Affiliations:** Department of Educational Science, School Pedagogy, Greifswald University, Greifswald, Germany

**Keywords:** teacher support, peer support, helplessness, ability to cope, academic achievement, multilevel analysis

## Abstract

According to the conservation of resources theory, social support provides resources to help overcome challenges. Although some empirical findings have emphasized the pivotal role of teacher support and/or peer support for students’ stress and academic achievement, multilevel analyses that consider contextual class and individual student effects are scarce. The current study addresses this gap and further includes gender, socio-economic status, and neuroticism as covariates. Multilevel analyses in Mplus were conducted. All measures were taken at the student level and then aggregated to the classroom level to estimate class-level relationships. Results revealed that on the individual level, teacher support was related to higher ability to cope and lower levels of helplessness, while on the class level, peer support by classmates was related to higher ability to cope and academic achievement. The context effects also show that in classes with higher peer support, students are more likely to benefit in terms of coping ability and achievement, whereas in classes with higher teacher support, students tend to show less coping ability.

## Introduction

Social relationships with peers and teachers play a pivotal role for students’ stress and learning outcomes as they act as resources that support learning and mitigate feelings of stress (Cohen et al., 1983; Hobfoll et al., 1990; Wentzel et al., 2017). The conservation of resources theory (COR) states that “human beings’ primary motivation is to build, protect, and foster their resource pools in order to protect the self-bond and the social bond that support the self.” (Buchwald and Schwarzer, 2010, p. 500). Unlike previous stress theories that focus on individual appraisals of stressors, COR assumes that stress has “central environmental, social, and cultural bases in terms of the demands on people to acquire and protect the circumstances that ensure their well-being and distance themselves from threats to well-being.” (Hobfoll and Ford, 2007, p. 565). Hence, the perception of stress is determined to a great extent by the social environment which is linked to the protection, gain, or loss of individual resources when faced with challenges (Hobfoll and Ford, 2007). COR can be applied to complex learning situations, e.g., the school context in which peers and teachers shape students’ social environment. Hence, if these relationships are perceived as supportive, students are less likely to experience stress and can invest their actual resources in the learning process which most likely increases their academic success. In contrast, if students feel a lack of support by peers and teachers, they consequently must invest more resources to handle and overcome stressful situations and be academically successful (see Hobfoll et al., 1990). In the current study, we define perceived stress along two dimensions, including helplessness and the ability to cope (Klein et al., 2016). Thereby, perceived helplessness reflects an individuals’ reaction to stress, whereas ability to cope emphasizes the self-assessed capability to cope with stressors (Roberti et al., 2006). Hence, if students feel helpless in stressful situations and exhibit low abilities to cope with stressors, chances of increased stress are high. As stress has become prevalent, particularly among the student cohort of young adolescents (Inchley et al., 2016) and presents a risk for students’ personal and academic development, it is important to investigate and detect factors that are part of students’ immediate environment and promise to mitigate feelings of stress.

A promising factor in reducing stress and boosting students’ academic achievement involves students’ relationships with teachers and peers at school. Teachers’ support can be quite complex and has been viewed as a multidimensional construct consisting of emotional, instrumental, informational, and feedback-related components (Tardy, 1985). Recent empirical studies show teacher-student relationships include emotional support, classroom organization, and instructional support (Downer et al., 2015; Hoferichter et al., 2020). Emotional support is characterized by emotional closeness, recognition, and interest for students’ concerns (Hamre and Pianta, 2006), whereas classroom organization includes, e.g., managing the teaching environment, student activities, and providing an orderly and functional classroom setting for students to achieve their educational goals (Creemers, 1994; Savage and Savage, 2009). Instructional support includes, e.g., helping students solve assignments, motivating students, and providing feedback on students’ learning progress (Kilpatrick Demaray et al., 2010).

Peer support describes the process of giving and receiving help from a similar person (with whom one shares similar demographics or social aspects), expressing empathy, encouragement, and support within a reciprocal relationship (Mead et al., 2001; Shalaby and Agyapong, 2020). As adolescents mature, social relationships change as they increasingly look to peers for support (Tarrant, 2002; Branje, 2018).

In sum, to capture social support, we speak of supportive relationships when they are characterized by close ties, care, esteem, and provide help if necessary (Sarason and Sarason, 2009). In this study, teacher support is conceptualized as the average of emotional and instructional support, thereby peer support relates to the positive relationship students have with their classmates. Students’ academic achievement is conceptualized by students’ grades in the subjects German, Math, and English.

So far, there have been some empirical studies investigating the single paths on how peer support and/or teacher support relate to students’ stress, while others have focused on how social support relates to students’ achievement (e.g., Tennant et al., 2015; Hoferichter and Raufelder, 2021; Hoferichter et al., 2021a). Although COR provides a framework for how social support may be related to student stress and academic achievement, it has not been empirically quantified how peer support as well as teacher support relate to students’ stress and academic achievement on an individual (student) and contextual (classroom) level. Educational systems such as schools are multilevel systems (Kozlowski and Klein, 2000) in which students are grouped into classes and share similar experiences. At the class level, perceived peer and teacher support constitute the class climate, which, when analyzed, must be treated as a class-level construct (Lüdtke et al., 2009; Bardach et al., 2020). The classroom climate is a common characteristic that all students in a class are exposed to. In empirical studies, such as this study, students are asked to indicate their perceptions of classroom climate, which consists of interpersonal communication and interactions between students and teachers. The use of multilevel analyses has the potential to identify the effects of a supportive classroom-level climate beyond the level of individual students.

### Teacher support, students’ stress, and academic achievement

Previous multilevel studies suggest that teacher support has a positive impact on student learning and behavioral outcomes. For example, Ma et al. (2021) found that perceived teacher support promoted the academic self-concept and enjoyment of learning, while Yildirim (2012) found a positive relationship between teacher support and students’ use of learning strategies in mathematics. Another multilevel study highlighted the important role of teacher support in student motivation and engagement (Wentzel et al., 2017). These multilevel studies take a promising approach by viewing classrooms as complex learning environments, taking into account individual (student) perspectives and contextual (classroom) aspects. However, when it comes to teacher support, student stress, and achievement, multilevel analyses are scarce, and empirical findings are limited to correlational or longitudinal studies that do not account for student clustering in classrooms.

Investigating into the relationship of teacher support and students’ stress, Hoferichter and Raufelder (2021) found that teacher support buffered the development of students’ academic exhaustion–a symptom of stress and burnout–over 1 school year. In a sample of elementary school students, it has been found that a positive teacher-student relationship serves children in their stress regulation measured by the stress hormone cortisol (Hughes, 2012). Students who rated their relationships with teachers as supportive exhibited the most optimal cortisol profiles and as such appropriately down-regulated stress compared to students with a lack of support from teachers.

Next, to the impact on students’ stress, supportive teacher-student relationships present an educational asset throughout students’ school career as they directly relate to students’ academic achievement and moreover to students’ behavioral variables that are linked to academic achievement. Empirical research indicates that students who perceive their teachers as supportive show better school adjustment (Sabol and Pianta, 2012), invest more in learning (Vansteenkiste et al., 2005), are more curious to learn new things (Hoferichter et al., 2020), and exhibit higher great point average (Tennant et al., 2015).

A wide range of empirical research that investigated the impact of teacher support on children’s’ academic achievement focused on elementary school students, while studies with secondary school students are underrepresented. This situation is particularly problematic, as studies have indicated that stress is a major problem for students during adolescence (Inchley et al., 2016). In their 3-year longitudinal study with elementary students at risk, Hughes et al. (2008) found that supportive relationships with teachers predicted students’ effortful engagement which further impacted their math and reading performance positively.

### Peer support, students’ stress, and academic achievement

During adolescence, peers become increasingly important as peer relationships contribute to social, emotional, and cognitive development (see Tarrant, 2002; Reitz et al., 2014). Although there are only a few studies that have investigated the direct link between peer support and students’ stress, various studies emphasize the beneficial role of supportive peer relationships for students’ mental and physical health (Rageliené, 2016), including better psychological well-being (Holt et al., 2018; Moore et al., 2018; Hoferichter et al., 2021a), adaptive behavior (La Greca and Harrison, 2005; Yeung and Leadbeater, 2010), and low levels of stress (Lyons and Jiang, 2021). Research suggests that peer support acts as a protective factor against depression, social anxiety (La Greca and Harrison, 2005), and test anxiety (Hoferichter and Raufelder, 2015). Examining classroom climate in a meta-analysis, Wang et al. (2020) found that classroom climate was negatively associated with students’ socioemotional distress. On a neurobiological level, Telzer et al. (2015) detected that peer support helped students regulate their response to stressors. Meanwhile, social exclusion by peers is related to disturbed neurodevelopment (Raufelder et al., 2021). In their review, Suresh et al. (2021) list the few studies that have investigated peer support as resource and summarize that in general, peer support has shown to improve the mental and physical health of students, including students’ stress and burnout, although literature, and particularly multilevel approaches, is limited.

Investigating peer support and students’ academic achievement, most studies disregard the multilevel structure of the educational context. Only a few multilevel studies have been conducted and indicate that peer support positively relates to students’ academic achievement (Burke and Sass, 2013; Wentzel et al., 2017). Burke and Sass (2013) found significant effects of peer support on students’ academic achievement only at the class but not individual level, indicating that the experience of peer support within the shared classroom context contributes to students’ achievement. In their study, Wentzel et al. (2017) examined peer support and learning effort at the individual student level and found positive associations, suggesting that emotional support facilitates learning. In their meta-analysis, Wang et al. (2020) find that overall classroom climate is associated with academic achievement. Single-level studies on the topic support the notation that peer support relates to increased academic involvement (Vargas-Madriz and Konishi, 2021) and academic achievement in Chemistry (Uzezi and Deya, 2017) and helps students to pursuit their academic goals (Patrick et al., 2004; Wentzel, 2005).

### The current study and hypotheses

Previous studies that investigated teacher and/or peer support emphasize the beneficial effect for students’ stress and/or academic achievement. From a theoretical perspective, supportive relationships act as resources that help to manage and overcome challenges that require more resources to consequently ensure the well-being of the individual (COR, Hobfoll and Ford, 2007).

However, to the best of our knowledge, no studies have investigated both teacher support and peer support simultaneously in relation to stress and academic achievement in one model, although both teachers and peers are part of students’ social environment at school, shaping the class climate. Furthermore, to evaluate the role of teacher and peer support for students’ stress (helplessness, ability to cope) and academic achievement (final grades in German, math, English) and consider students’ classroom context, it is necessary to (a) include both support variables as predictors for students’ stress and academic achievement in one statistical model as well as (b) apply a multilevel model to identify individual and contextual effects – which are the aims of the current study.

Students in class are usually interdependent with their peers which means that they influence each other and share a similar context, e.g., same teachers, same classroom settings, and rules, which distinguishes them from students that attend different classrooms. Therefore, it may be beneficial to examine the individual’s experience of support by teachers and peers related to stress and academic achievement considering the classroom context by means of multilevel analyses (Kozlowski and Klein, 2000; Bardach et al., 2020).

Based on the outlined research and COR, we hypothesize the following:

*H1*: Individual students who experience teacher support and/or peer support are more likely to cope with stressors and are less likely to report helplessness. In addition, those students also exhibit higher academic achievement.

*H2*: The average teacher and peer support in class relates to student stress perception and academic achievement. As previous analysis on the topic in light of contextual and individual effects are scarce, we follow an exploratory approach.

As students’ gender, socio-economic status as well as the personality trait neuroticism have been linked to students’ stress and academic achievement, they were included as covariates into the model. In detail, girls tend to report higher stress levels (Salmela-Aro et al., 2009; Hoferichter et al., 2021b) and exhibit higher academic achievement (Voyer and Voyer, 2014) compared to boys. Furthermore, students with lower socio-economic status tend to experience more stress (Roubinov et al., 2018; Tarullo et al., 2020) and exhibit lower academic achievement (Sirin, 2005). Neuroticism was included in the analysis, as it is related to higher threat appraisals (Schneider, 2004) and an intensified stress reactivity (Suls, 2001) which may compromise academic achievement (Hakimi et al., 2011).

## Materials and methods

### Participants

The dataset used in this study is built on a large, quantitative questionnaire survey of German adolescent students in Mecklenburg-Western Pomerania. The data were collected from 11 randomly selected secondary schools during the winter term 2018/2019 of the German school year. Schools that were located less than 2 h away from the research facility were contacted and invited to participate in the study. The 11 participating schools represent about 73% of all schools contacted of which all were located in urban areas. A total of 733 7th and 8th grade students (*M_age_* = 13.97, *SD* = 0.41, 52% girls) participated in the questionnaire. They belonged to 60 classes. For the variables used in the study, the average cluster size varied between 11.60 and 12.23 (6.04 ≤ *SD* ≤ 6.34). As the state of Mecklenburg-Western Pomerania has only a small proportion of ethnic minority residents (4.3%; Statistisches Amt Mecklenburg-Vorpommern, 2018), data on ethnic background were not collected as the anonymity of the participants could become compromised.

### Procedure

To comply with ethical standards (American Psychological Association, 2002), a strict procedure was followed prior to all data collection. First, permissions were obtained from the respective educational authorities (Ministry for Education, Science and Culture Mecklenburg-Western Pomerania). Second, informed consent and permissions were consecutively obtained from schools, parents, and students. The students were informed in written and orally about the nature of the study and its goals, the voluntary nature of participation as well as the assurance of anonymity of data collection. At least two trained research assistants were present throughout the data collection. They explained the instruments to the students and particularly, how to use the Likert scales. Furthermore, the research assistants answered any comprehension questions.

### Measures

#### Teacher and peer support in class

Teacher and peer support in class were assessed with two subscales by Torsheim et al. (2000). Both subscales consist of five items each with answers ranging from 1 (“not true at all”) to 5 (“completely true”). They evaluate students’ satisfaction regarding the support from teachers and peers in the classroom, as well as the availability of support and helpfulness (e.g., “Our teachers treat us fairly,” “The students in my class enjoy being together”). The teacher support scale exhibited good internal reliability (α = 0.71) as did the peer support in class scale (α = 0.78).

#### Perceived stress

Perceived stress was evaluated with the help of the German version of the *Perceived Stress Scale* (Klein et al., 2016) which was originally developed by Cohen et al. (1983). The scale consists of a two-dimensional structure with two related subscales. Both subscales consist of five items each and were measured on a five-point Likert scale from 1 (“never”) to 5 (“very often”). The subscale perceived helplessness refers to a general measurement of stress as it emphasizes individual reactions to stress (e.g., “In the last month, how often have you been upset, because of something that happened unexpectedly?”). The scale exhibited a very good internal consistency of 0.81. The subscale perceived ability to cope relates to an individual’s assessment of the ability to cope with stressors (e.g., “In the last month, have you felt that you were unable to control the important things in your life?”). This subscale achieved a good internal reliability as well (α = 0.71).

#### Achievement

To measure achievement, the grade point average (GPA) was assessed by students’ self-reporting on their last report card in the three main subjects Math, German, and English. In Germany, the grade scale usually ranges from “1″ (best outcome possible) to “6″ (worst outcome possible).

#### Covariates

To rule out potential confounders for perceived stress and academic achievement, we included several covariates in our analyses. For the socio-economic status, we used the “book question” (Nachtigall and Kröhne, 2004) and asked the students about the number of books that are available in their households (e.g., “How many books do you have at home?”). Answers were measured on a 5-point Likert scale ranging from 1 (“any to few books”) to 5 (“over 200 books”). Moreover, neuroticism was assessed with the help of a subscale from the *Big Five Kids Inventory* (Bleidorn and Ostendorf, 2009), which is based on the scales developed by Mervielde and De Fruyt (1999). The two items (e.g., “I doubt myself”) were measured on a 5-point Likert scale ranging from 1 (“hardly”) to 5 (“very”). The scale exhibited a good internal consistency of α = 0.77. Gender was also included as covariate with 0 = boys and 1 = girls.

## Statistical analyses

The statistical analyses were conducted using Mplus 8.1 (Muthén and Muthén, 1998-2017). All analyses were performed using robust maximum likelihood estimation and missing data were compensated for using the full information maximum likelihood approach.

As our data are hierarchically structured (i.e., students clustered in classes), we performed multilevel structural equation modeling (MLSEM) (Hox et al., 2018). This approach allows to differentiate effects on the student level from those on the class level. Multilevel analyses can be further extended to subsequent hierarchical structures (e.g., schools). However, the class level was chosen as it represents the immediate context of students’ learning environment (van Ewijk and Sleegers, 2010). Lüdtke et al. (2009) highlight in their study that using a multilevel approach is usually warranted when examining the impact of learning environment characteristics (i.e., teacher and classmate support). Ignoring the different levels (e.g., student vs. classroom level) leads to aggregated and biased parameter estimates (see also Marsh et al., 2009).

After careful consideration, we made use of parcels instead of single-item data for the scales’ indicators. Parceling is a technique widely used in psychology and social sciences to produce more stable results due to more parsimonious models (Little et al., 2002; Nasser and Wisenbaker, 2003). Accordingly, random parcels were built meaning that the scales’ items were randomly assigned to built one parcel. In case of perceived helplessness, three random items were assigned to two parcels. For all other variables, two random items were assigned, so that each latent variable would be measured by two parcel indicators. Parceling has several advantages over item-level data. The advantages relate to psychometric characteristics, such higher reliability and a higher ratio of common-to-unique factor variance. In terms of model estimation, parceling has a lower likelihood of distributional violations and it leads to a more parsimonious model with fewer parameter estimates, a lower likelihood of correlated residuals and cross-loadings, and reduced sources of sampling error (Little et al., 2002, 2013). Achievement, neuroticism, gender, and SES were entered as manifest variables.

To test our hypotheses whether teacher as well as peer support would be related to lower stress levels and higher academic achievement among secondary school students, a MLSEM was built. This MLSEM builds upon the work of Lüdtke et al. (2011) who presented the latent-measurement/manifest-aggregation approach. This approach is referred to as a partial correction approach, as it corrects bias in the estimates due to item sampling (latent measurement), but it does not correct the estimates for bias in the sampling of individuals (manifest aggregation). The latter indicates that classroom-level constructs are based on group average of individual-level constructs. However, this approach is preferably over the doubly latent approach (Marsh et al., 2009), if there is only limited information at the cluster level (e.g., few clusters or few individuals within certain clusters) (Lüdtke et al., 2011). Contrarily to our dataset, doubly latent models require at least 50 clusters (preferably 100) with 10–15 individuals within each cluster. As group differences were of utmost interest in this study, we used group-mean centering for the predictors at the student level. Thereby, only in-group variance is included in the prediction meaning that the regressions at L1 represent the expected change of an outcome variable based on the increase of one within-cluster unit in the predictor (Enders and Tofighi, 2007; Enders, 2013).

Accordingly, a null model was estimated first to confirm the factor structure of the latent constructs and to investigate their variances at the different levels (student level and class level). This separation of variance is necessary to compute the intra-class correlations (ICC). The ICC(1) provides information about potential individual variance at the two levels, whereas the ICC(2) provides an estimate of reliability of aggregated classroom mean ratings (Snijders and Bosker, 2012). Particularly, ICC(1) is necessary to investigate the amount variance at L2 that can be analyzed by adding predictors at the respective levels. This examination was necessary to determine whether a multilevel approach is actually warranted for our data. To be precise, only if there were substantial differences in the dependent variables (achievement, ability to cope, and perceived helplessness), a multilevel approach should be favored over a single-level model. Subsequently, this model was extended with L1 predictors (model 1) and finally with L1 and L2 predictors (model 2; teacher support, classmate support, SES, gender, and neuroticism).

Additionally, we added parameters to the analyses that computed the context-effects. A context effect is present, if an aggregated variable at class level is still associated with the dependent variable after controlling for the same effect on the individual level. Consequently, there are context effects if the slopes of the within-group regressions are different from the between-group regressions (Raudenbush and Bryk, 2002). This difference between both regressions was therefore added as an additional parameter in the *model constraint* option of Mplus and was further standardized to facilitate interpretation. The standardization is based on multiplying the unstandardized effect with two standard deviations of the predictor variable at L2 divided by the total variance of the L1 dependent variable. The standardized effect size can then be interpreted as the difference in the dependent variable between two L2 clusters that differ by two standard deviations on the predictor variable (Marsh et al., 2009).

Evaluations of the model fit are based on the recommendations of Hu and Bentler (1999): Consequently, we report and evaluate χ^2^ test of model fit, Comparative-Fit-Index (CFI), Tucker-Lewis Index (TLI), standardized root mean square Residual (SRMR), and root mean square error of approximation (RMSEA) with its 90% confidence intervals.

## Results

Table 1 shows the manifest zero-order correlations of the study’s variables and Table 2 their descriptive statistics.

**Table 1:** Zero-order correlation coefficients among all study variables at student and classroom level.

	1	2	3	4	5	6	7	8
1. Helplessness		−0.73***	−0.30	−0.36*	−0.07	−0.48**	0.90***	0.54***
2. Ability to cope	−0.48***		0.52**	0.70***	−0.01	0.82***	−0.78***	−0.92***
3. Teacher support	−0.23***	0.18***		0.77***	−0.05	0.40*	−0.26	−0.56***
4. Peer support	−0.18***	0.15***	0.34***		−0.24	0.56***	−0.18	−0.74***
5. Gender	−0.18***	0.21***	−0.06	−0.08*		−0.36	−0.02	0.28
6. SES	−0.01	0.04	−0.01	−0.01	−0.06		−0.47	−0.97***
7. Neuroticism	0.54***	−0.42***	−0.11**	−0.07	−0.36***	0.01		0.52*
8. GPA	−0.21***	0.21***	0.07	0.03	−0.17***	0.14***	0.02	

Lower triangle = L1; upper triangle = L2; gender: (0 = girls, 1 = boys). Estimates are significant at *p < 0.05, **p < 0.01, ***p < 0.001.

**Table 2:** Descriptive statistics.

	Range	*M*	*Var_within_*	*Var_between_*	*Skewness*	*Kurtosis*	*ICC(1)*	*ICC(2)*
1. Helplessness	1–5	2.80	0.47	0.07	0.07	−0.42	0.13	0.64
2. Ability to cope	1–5	3.22	0.39	0.09	−0.25	−0.04	0.18	0.73
3. Teacher support	1–5	3.59	0.39	0.05	−0.67	1.10	0.11	0.61
4. Peer support	1–5	3.92	0.41	0.12	−1.07	1.69	0.23	0.78
5. Gender	0–1	0.47	0.24	0.01	0.09	−2.00	0.05	0.41
6. SES	1–5	3.39	1.30	0.40	−0.35	−0.98	0.24	0.78
7. Neuroticism	1–5	2.86	1.24	0.06	0.12	−85	0.05	0.37
8. GPA*	1–6	2.57	0.32	0.21	0.35	−0.11	0.40	0.89

### Multilevel structural equation modeling

Initially, we conducted a null model in which only the dependent variables were modeled. Similarly to the ICC(1) values, this model served as a reference model to examine whether there is significant variance of the dependent variables at both levels. The null model showed a good fit [χ^2^(10) = 18.89, *p*(χ^2^) < 0.05; CFI = 0.99, TLI = 0.98, SRMR_within_ = 0.02, SRMR_between_ = 0.07, RMSEA = 0.07]. At the student level, all three dependent variables exhibited significant variances: perceived helplessness (σ^2^ = 0.38, *p* < 0.001), ability to cope (σ^2^ = 0.24, *p* < 0.001), and achievement (σ^2^ = 0.35, *p* < 0.001). Similarly, all variances at the between level were significant, thus warranting a multilevel approach: perceived helplessness (σ^2^ = 0.06, *p* < 0.01), ability to cope (σ^2^ = 0.08, *p* < 0.001), and achievement (σ^2^ = 0.18, *p* < 0.001).

Subsequently, we added predictors based on theory and prior empirical research to L1 (model 1). This model achieved an adequate fit: χ^2^(39) = 103.942, *p*(χ^2^) < 0.001; CFI = 0.97, TLI = 0.94, SRMR_within_ = 0.03, SRMR_between_ = 0.30, RMSEA = 0.05 (see Table 3).

**Table 3:** Results of multilevel structural equation modeling.

Coefficient	Ability to cope				Helplessness				Achievement			
	*B*	*SE*	*p*	β	*B*	*SE*	*p*	β	*B*	SE	*p*	β
Level 1 - Student												
teacher support	**0.18**	**0.07**	**= 0.02**	**0.17**	**−0.23**	**0.07**	**< 0.01**	**−0.18**	0.08	0.07	= 0.23	0.06
peer support	0.10	0.07	= 0.14	0.10	−0.14	0.09	= 0.11	−0.11	−0.01	0.06	= 0.92	−0.00
neuroticism	**−0.34**	**0.03**	**< 0.001**	**−0.45**	**0.34**	**0.03**	**< 0.001**	**0.59**	**−0.08**	**0.03**	**< 0.01**	**−0.12**
Gender	**0.13**	**0.06**	**= 0.04**	**0.12**	−0.03	0.06	= 0.60	−0.02	**−0.20**	**0.07**	**< 0.01**	**−0.14**
SES	**0.08**	**0.02**	**< 0.001**	**0.19**	**−0.05**	**0.02**	**= 0.02**	**−0.10**	**0.19**	**0.03**	**< 0.001**	**0.33**
*R^2^_within_*				**0.37**				**0.48**				**0.15**

Bold values indicate significance.

**Figure 1:**
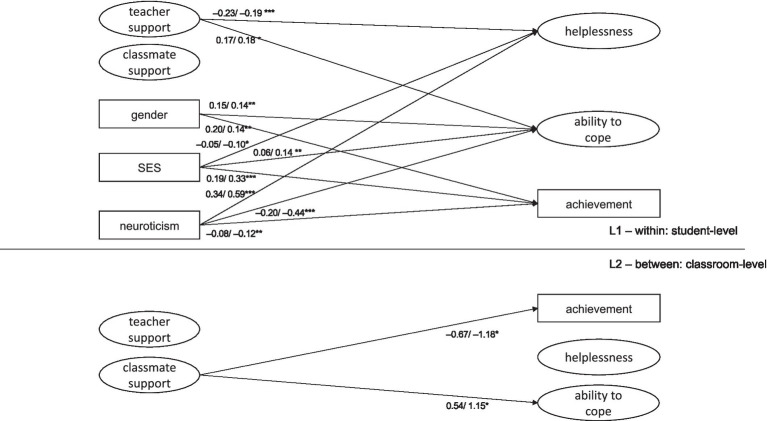
Final multilevel structural equation model. Note. Only significant paths are shown. Estimates are shown as first unstandardized and second as standardized estimates at *p < 0.05, **p < 0.01, ***p < 0.001.

Further, we added predictors to L2 which resulted in our final model (model 2; Figure 1; Table 3). This final model achieved a good fit: χ^2^(54) = 94.90, *p*(χ^2^) < 0.001; CFI = 0.98, TLI = 0.96, SRMR_within_ = 0.03, SRMR_between_ = 0.11, RMSEA = 0.03. In this model, the significant paths of model 1 remained robust in light of the addition of the added predictors at L2. However, as indicated by the R^2^ values, significant proportions of variance were explained by classroom differences of the predictors at L2 (see Table 4).

**Table 4:** Results of multilevel structural equation modeling.

Coefficient	Ability to cope				Helplessness				Achievement			
	*B*	*SE*	*p*	β	*B*	*SE*	*p*	β	*B*	SE	*p*	β
Level 1 - Student												
teacher support	**0.17**	**0.07**	**= 0.01**	**0.18**	**−0.23**	**0.07**	**< 0.001**	**−0.19**	0.07	0.06	= 0.23	0.05
peer support	0.11	0.07	= 0.10	0.11	−0.14	0.08	= 0.08	−0.11	0.00	0.06	= 0.95	0.00
neuroticism	**−0.20**	**0.02**	**< 0.001**	**−0.44**	**0.34**	**0.03**	**< 0.001**	**0.59**	**−0.08**	**0.03**	**< 0.01**	**−0.12**
Gender	**0.15**	**0.06**	**< 0.01**	**0.14**	−0.03	0.06	= 0.61	−0.02	**−0.20**	**0.07**	**< 0.01**	**−0.14**
SES	**0.06**	**0.02**	**< 0.01**	**0.14**	**−0.05**	**0.02**	**= 0.03**	**−0.10**	**0.19**	**0.03**	**< 0.001**	**0.33**
*R^2^_within_*				**0.37**				**0.48**				**0.15**
Level 2 - Class												
teacher support	−0.27	0.16	= 0.10	−0.48	0.01	0.19	= 0.96	0.02	0.14	0.36	= 0.69	0.09
peer support	**0.54**	**0.15**	**< 0.001**	**1.15**	−0.12	0.17	= 0.48	−0.29	**0.66**	**0.25**	**< 0.01**	**0.49**
*R^2^_between_*				**0.79**				0.08				**0.31**
Additional parameters												
context-effect teacher support	**−0.45**	**0.19**	**= 0.02**	**−3.39**	0.24	0.20	= 0.22	1.00	0.07	0.38	= 0.86	0.10
context-effect peer support	**0.43**	**0.17**	**= 0.01**	**3.97**	0.02	0.18	= 0.91	1.00	**0.67**	**0.26**	**= 0.01**	**1.18**

Significant effects are displayed in bold at the 0.05 level. Gender: (0 = girls, 1 = boys).

Standardized factor loadings of the latent constructs ranged between 0.64 and 0.81 on the within level and between 0.75 and 1.00 on the between level. The model included significant correlations of the predictor variables: gender was significantly associated with peer support (*r =* −0.12, *p* < 0.05) and neuroticism (*r =* −0.35, *p <* 0.001). Moreover, teacher support was significantly associated with neuroticism (*r* = −0.14, *p* < 0.01) and peer support (*r* = 0.46, *p* < 0.001). Lastly, ability to cope was associated with perceived helplessness (*r* = −0.41, *p* < 0.001). On the between level, average peer support was significantly associated with teacher support (*r* = 0.70, *p* < 0.001).

At the student level, teacher support predicted perceived helplessness (*B* = −0.23, β = −0.19, *SE = 0*.06*, p < 0.*001) and ability to cope (*B* = 0.17, β =0.18, *SE = 0*.07*, p < 0.*05). Thus, if the students in our study perceived their teachers to be supportive, students indicated less perceived helplessness and more ability to cope. Moreover, gender proved to be a significant covariate, indicating that boys have a higher ability to cope (*B* = 0.15, β = 0.14, *SE = 0*.06*, p < 0.*01) and exhibited lower achievement than girls (*B* = −0.20, β = −0.14, *SE* = 0.07, *p* < 0.01). Moreover, neuroticism negatively predicted ability to cope (*B* = −0.20, β = 0.44, *SE = 0*.02*, p < 0.*001), positively predicted perceived helplessness (*B* = 0.34, β = 0.59, *SE = 0.*03*, p < 0.*001), as well as academic achievement (*B* = −0.08, β = −0.12, *SE* = 0.03, *p* < 0.01). Moreover, SES was significantly related to all three dependent variables: the higher students’ SES, the more likely they exhibited higher ability to cope (*B* = 0.06, β = 0.14, *SE* = 0.02, *p* < 0.01), lower perceived helplessness (*B* = −0.05, β = −0.10, *SE* = 0.02, *p* < 0.05), and higher achievement (*B* = 0.19, β = 0.33, *SE* = 0.03, *p* < 0.001).

On the classroom level, average peer support by classmates significantly predicted class average ability to cope (*B* = 0.54, β = 1.15, *SE = 0*.15*, p < 0.*001). Additionally, average classmate support significantly predicted average achievement (*B* = 0.66, β = 0.49, *SE* = 0.25, *p* < 0.01).

Three contextual effects were found to be significant: (1) the association between peer support in class and ability to cope (*B =* 0.43, β = 3.97, *SE =* 0.17, *p* = 0.01) meaning that if two students who indicate equal values on peer support, the one being in a classroom with a higher average of peer support would perceive significantly more ability to cope. (2) the association between teacher support and ability to cope (*B* = −0.45, β = −3.39, *SE* = 0.19, *p* < 0.05) meaning that if two students who indicate equal values on teacher support, the one being in a classroom with a higher average of teacher support would perceive significantly less ability to cope. Lastly, (3) the association between peer support and academic achievement (*B* = 0.67, β = 1.18, *SE* = 0.26, *p* < 0.05) meaning that if two students who indicated equal values on peer support in class, the one being in a classroom with a higher average peer support would exhibit higher academic achievement.

The model explained 48% (*R^2^* = 0.48, *p* < 0.001) of variation of perceived helplessness, 37% of variation of ability to cope (*R^2^* = 0.37, *p* < 0.001), and 15% of variation of students’ academic achievement (*R^2^* = 0.15, *p* < 0.001) on the student level. On the classroom level, the model explained 79% (*R^2^* = 0.79, *p* < 0.001) of variation of class average ability to cope, 8% (*R^2^* = 0.08, *p* = 0.51) of variation of class average perceived helplessness, and 31% (*R^2^* = 0.31, *p* < 0.001) of variation of classes’ average academic achievement.

## Discussion

The current study investigated how perceived teacher and peer support in class relate to secondary school students’ stress, captured by ability to cope and helplessness, as well as students’ academic achievement on both the individual and the class level. Because teacher and peer support shape the class climate, which is a class-level variable, multilevel analyses were applied to detect individual student and contextual classroom effects, including gender, SES, and neuroticism as control variables.

The theoretical underpinnings of the study include COR (Hobfoll et al., 1990; Hobfoll and Ford, 2007), which assumes that social support acts as a resource during challenges. Specifically, the investment of resources is required to successfully face and overcome challenges. Applying COR to the school context and the current study, we expected that peer and teacher support would provide resources to help students manage their stress and improve their academic performance.

The multilevel analyses partly confirmed H1 by revealing that on an individual student level, teacher support was related to higher ability to cope and lower levels of helplessness. Hence, if a student perceives teachers as supportive, this student experiences less stress, as he/she applies coping strategies to deal with stressors and reports lower helplessness. These findings are in line with previous correlational and longitudinal studies that investigated the direct paths of the predictor variables teacher support (Hughes, 2012; Hoferichter and Raufelder, 2021) for students’ stress level. Contrary to H1, however, no significant relationship was found between teacher support and grades. This contradicts previous studies, possibly because they are all based on data from elementary school students (Ladd and Burgess, 2001; Hughes et al., 2008; Mason et al., 2019). In general, teacher-student relationships are perceived as more supportive and caring in elementary schools, and the type of teaching (e.g., subject teachers; teacher-centered learning) also differs greatly in the two types of schools (Wigfield et al., 1991; Anderman and Maehr, 1994; Midgley et al., 1995; Anderman and Midgley, 1997). In addition, the results of previous studies may differ due to different operationalization procedures.

H1 could also not be confirmed in the sense that no significant associations between perceived peer support and stress experience or grades were found at the individual level, which contradicts previous studies (Burke and Sass, 2013; Uzezi and Deya, 2017; Vargas-Madriz and Konishi, 2021). Interestingly, and in line with H2, peer support within the classroom had a significant association with ability to cope and academic achievement, when analyzed on the class level. Hence, when peer support was aggregated on a class level and as such class context taken into consideration, students reported higher ability to cope in stressful situations and better GPA, when they perceived peer support by their classmates. In line with these results, Burke and Sass (2013) did also find that peer support was related to higher students’ academic achievement only on the class level. The results reveal that classmates together present a powerful context providing support to their peers which in turn is related to better coping strategies in stressful situations and better academic performance. Meanwhile, teachers who support their students may be able to help them cope with stressors and feel less helpless.

The current study also partly confirmed H2 as it was found that students’ perceived class context was related to the degree students were able to cope with stress and be academically successful. Context effects can be interpreted as a comparison between two identical students in different classes (contexts). In detail, if two students who indicate equal values regarding peer support, the one being in a classroom with a higher average of peer support would perceive significantly more ability to cope and higher academic achievement. Furthermore, the current study also revealed that students who are part of a context in which teachers are perceived as supportive tend to exhibit less ability to cope. This finding may be counterintuitive, as COR and previous studies suggest that teacher support is related to lower stress in students (Hughes, 2012; Hoferichter and Raufelder, 2021). Therefore, this finding could lead to the conclusion that high levels of teacher support affect students’ coping skills, as excessive support can undermine self-development. Perhaps, students in classes with very high teacher support do not feel the need to expand their coping skills because the high teacher support cancels out their stressful experience. When teachers provide too much support, they can interfere with students’ autonomy and competence, which are important prerequisites for developing self-determined behaviors and skills (Catalano et al., 2004; Wehmeyer, 2005). Self-determined behavior refers to “volitional actions that enable one to act as the primary causal agent in one’s life and to maintain or improve one’s quality of life” (Hui and Tsang, 2012, p. 117). In other words, students who have the opportunity to experience autonomy and competence are more likely to develop self-determined behaviors, which, in turn, can strengthen their coping skills. Future studies, however, should examine the varying degrees of teacher support from the perspective of students in order to differentiate how much support teachers should provide to help students cope with stressors.

Considering the covariates that were included in the model on the student level to rule out potential confounders, it was found that neurotic students reported less ability to cope and more helplessness as well as worse GPA compared to non-neurotic students. As neurotic individuals tend to experience higher threat appraisals and are more vulnerable to stress which compromises their academic achievement, the current studies’ findings are in line with previous research (Suls, 2001; Schneider, 2004; Hakimi et al., 2011).

Furthermore, students from high socio-economic backgrounds reported higher ability to cope and less helplessness as well as better academic achievement compared to students from lower socio-economic backgrounds. This finding is in line with previous studies, indicating higher stress levels among low SES students (Roubinov et al., 2018; Tarullo et al., 2020) as well as medium to strong SES-achievement relations (Sirin, 2005). As expected, girls reported lower ability to cope with stressors and better GPA compared to boys, which was also found by Hoferichter et al. (2021b) and Salmela-Aro et al. (2009) as well as Voyer and Voyer (2014), respectively.

In sum, the current study emphasizes the essential role of teachers and peers for students’ stress management and academic achievement. The multilevel approach allowed us to identify different effects at the individual and class levels: While on the individual level particularly teacher support was found to be positively related to students’ stress management and academic achievement, on the class level and considering context effects, peer support related to students’ ability to cope with stressors and to high academic achievement. In other words, general class climate characterized by mutual support is needed above all to reduce the experience of stress and have a positive effect on academic performance. Thereby, the role of teachers differs from peer support, as individual students who perceive their teachers as supportive exhibit better stress management in general, i.e., high ability to cope and low helplessness. On the other hand, if all teachers in a class are perceived as highly supportive, there may be a reversal effect insofar as students then tend to report fewer coping skills. By considering the hierarchical structure of students nested in classrooms, this study could give even more detailed information on how teacher and peer support relate to students’ stress and academic ability. This study reveals empirical findings that contribute to research on social resources in the frame of the conservation of resource theory (Hobfoll et al., 1990), revealing that students’ stress and academic achievement to a large part are related to the quality of teacher and peer support differently on the individual and class level. Thus, while COR provides a general approach to the function of social resources as protective factors in difficult situations, our empirical study provides additional information on how classroom climate variables differentially affect student stress and academic achievement, illustrating the complex nature of social relationships and their impact on student outcomes.

Transferring the findings to the school context, school staff should be advised that their 1:1 relationship with students enhances students’ ability to deal with challenging situations and enables them to take action rather than feeling helpless. Thus, a teacher who responds to the student individually, attends to the student’s concerns and interests, and expresses a great deal of appreciation to the student plays an important role in helping the student cope with stress. Because peer support as a classroom variable plays an important role in students’ coping strategies and academic achievement, teachers can consider peers as significant protective factors that promote their classmates’ academic achievement. Collaborative classroom activities, shared learning scenarios, and peer feedback should be integrated into daily classroom routines (see Simonsmeier et al., 2020). In addition, school staff should understand their role in the classroom as mentors who guide learning processes while keeping a low profile rather than overemphasizing their support for students, as too much teacher support can hinder students’ personal and academic development.

### Strengths, limitations, and future research

This study examined how both teacher and peer support relate to students’ stress and academic achievement by considering covariates such as gender, SES, and neuroticism. Thereby, this study investigates social resources from the immediate environment of students that can further benefit interventional programs that aim at reducing stress and increasing academic success among students. A strength of this study is the multilevel analysis that considers individual and context effects in the interplay of the variables of interest. However, as in all empirical studies, there are limitations that have to be taken into consideration when interpreting the results, such as the cross-sectional nature of data investigated. Hence, no causal relationships between the variables may be derived from the analyses. Future research should therefore investigate the longitudinal relationship between teacher and peer support on students’ stress and academic achievement over the school years, to consider long-term effects of social resources and further develop COR by adding the time factor and by covering developmental processes of students. As the school context shapes students’ stress, school engagement, and motivation (Hoferichter and Raufelder, 2022), future studies are advised to investigate potential differences across students from various school types (e.g., lower- and higher track schools) and consider different age groups (e.g., elementary school students), as peer relationships and teacher-student relationships change during students’ school career as well as students’ needs for social support from different agencies (Tarrant, 2002; Branje, 2018; Hoferichter et al., 2021a). In addition to self-report data, future studies may include teacher and parental ratings when it comes to students’ ability to cope and helplessness as well as include competencies of students in various subjects that complement GPA.

As the current study indicates individual and group-level specifics with respect to the association of teacher and peer support for students’ stress and academic achievement, further person-oriented approaches promise to bring to light detailed information on the topic by addressing the following research questions: What would students’ profiles look like given varying degrees of teacher support, students’ coping skills, helplessness, and achievement? Would these profiles be stable across school years? How might different learning environments (e.g., teacher-centered instruction, self-directed learning) contribute to students’ coping skills and academic achievement? How do boys and girls differ in their need for peer and teacher support to develop their coping skills and succeed academically? How does differentiated teacher support, such as emotional and instructional support and classroom management, contribute to students’ stress development and academic achievement?

## Data availability statement

The raw data supporting the conclusions of this article will be made available by the authors, without undue reservation.

## Ethics statement

The studies involving human participants were reviewed and approved by Ethikkommission der Universitätsmedizin Greifswald. Written informed consent to participate in this study was provided by the participants’ legal guardian/next of kin as well as by students themselves.

## Author contributions

FH and SK designed the study. FH wrote the theoretical part and discussion, while SK did the statistical analyses and wrote the methods and results sections. DR acted as consultant and edited the MS. All authors contributed to the article and approved the submitted version.

## Funding

This research was funded by the “Anschubfinanzierung” of the University of Greifswald. We acknowledge support for the Article Processing Charge from the DFG (German Research Foundation, 393148499) and the Open Access Publication Fund of the University of Greifswald.

## Conflict of interest

The authors declare that the research was conducted in the absence of any commercial or financial relationships that could be construed as a potential conflict of interest.

## Publisher’s note

All claims expressed in this article are solely those of the authors and do not necessarily represent those of their affiliated organizations, or those of the publisher, the editors and the reviewers. Any product that may be evaluated in this article, or claim that may be made by its manufacturer, is not guaranteed or endorsed by the publisher.
