# Supplementary material for: How teacher and classmate support relate to students’ stress and academic achievement
Source: Front Psychol. 2022 Nov 28;13:992497. doi: 10.3389/fpsyg.2022.992497 (PMC9742244; doi:10.3389/fpsyg.2022.992497)
Supplement: Supplementary file 1 [file Table_1.DOCX]

**Appendix A: Mplus Syntax for the Null Model**

TITLE: NULL MODEL

DATA: FILE = "data.dat";

VARIABLE:

NAMES = class_id PS02_01 PS02_02 PS02_03 PS02_04 PS02_05 PS02_06 PS02_07 PS02_08 PS02_09 PS02_10 NN02_01 + NN02_02 + NN02_03 TC02_01 TC02_02 TC02_03 TC02_04 TC02_05 TC02_06 TC02_07 TC02_08;

MISSING= all(-99);

USEVAR =

HE1 HE2 CO1 CO2 ACH a_ACH;

WITHIN =

ACH;

BETWEEN =

a_ACH;

missing are all (-9,99);

CLUSTER= class_id;

DEFINE:

HE1 = mean(PS02_03 PS02_09 PS02_10);

HE2 = mean(PS02_02 PS02_01 PS02_06);

CO1 = mean(PS02_07 PS02_08);

CO2 = mean(PS02_05 PS02_04);

ACH = (NN02_01 + NN02_02 + NN02_03)/3;

a_ACH = cluster_mean(ACH);

ANALYSIS:

TYPE = TWOLEVEL;

ESTIMATOR = MLR;

PROCESS =2;

MODEL:

%within%

WHELP by

HE1 (1)

HE2 (2);

WCOPE by

CO1 (3)

CO2 (4);

WCOPE;

WHELP;

ACH WITH WHELP WCOPE;

%between%

BHELP by

HE1 (1)

HE2 (2);

BCOPE by

CO1 (3)

CO2 (4);

BCOPE;

BHELP;

A_ACH WITH BHELP BCOPE;

output: sampstat tech4 stdyx modindices (10);

**Appendix B: Mplus Syntax for the Final Model**

TITLE: FINAL MODEL

DATA: FILE = "data.dat";

VARIABLE:

NAMES = class_id gender PS02_01 PS02_02 PS02_03 PS02_04 PS02_05 PS02_06 PS02_07 PS02_08 PS02_09 PS02_10 NN02_01 + NN02_02 + NN02_03 BF02_01 BF02_02;

USEVAR =

gender

BU01

HE1

HE2

CO1

CO2

pTC1

pTC2

a_pTC1

a_pTC2

pCS1

pCS2

a_pCS1

a_pCS2

ACH

a_ACH

NEURO;

WITHIN =

NEURO

pTC1

pTC2

pCS1

pCS2

gender

BU01

ACH;

BETWEEN =

a_pTC1

a_pTC2

a_pCS1

a_pCS2

a_ACH;

MISSING = all (99);

CLUSTER= class_id;

DEFINE:

HE1 = mean(PS02_03 PS02_09 PS02_10);

HE2 = mean(PS02_02 PS02_01 PS02_06);

CO1 = mean(PS02_07 PS02_08);

CO2 = mean(PS02_05 PS02_04);

pTC1 = mean(TC02_01 TC02_02);

pTC2 = mean(TC02_03 TC02_04);

pCS1 = mean(TC02_05 TC02_06);

pCS2 = mean(TC02_07 TC02_08);

NEURO = (BF02_01+BF02_02)/2;

ACH = (NN02_01 + NN02_02 + NN02_03)/3;

a_pTC1 = cluster_mean(pTC1);

a_pTC2 = cluster_mean(pTC2);

a_pCS1 = cluster_mean(pCS1);

a_pCS2 = cluster_mean(pCS2);

a_ACH = cluster_mean(ACH);

CENTER

pTC1

pTC2

pCS1

pCS2(GROUPMEAN);

ANALYSIS:

TYPE = TWOLEVEL;

ESTIMATOR = MLR;

PROCESS = 2;

MODEL:

%within%

WTC by

pTC1 (1)

pTC2 (2);

WCS by

pCS1 (3)

pCS2 (4);

WHELP by

HE1 (5)

HE2 (6);

WCOPE by

CO1 (7)

CO2 (8);

WCOPE on WTC (b1_with);

WHELP on WTC (b2_with);

WCOPE on WCS (b3_with);

WHELP on WCS (b4_with);

ACH on WTC (b5_with);

ACH on WCS (b6_with);

WCOPE (CO_w);

WHELP (HE_w);

WTC (TC_W);

WCS (CS_W);

ACH (ACH_W);

WCOPE ON gender BU01 NEURO;

WHELP ON gender BU01 NEURO;

ACH ON gender BU01 NEURO;

gender BU01 NEURO WITH WTC WCS gender BU01 NEURO;

%between%

BTC by

a_pTC1 (1)

a_pTC2 (2);

BCS by

a_pCS1 (3)

a_pCS2 (4);

BHELP by

HE1 (5)

HE2 (6);

BCOPE by

CO1 (7)

CO2 (8);

BCOPE (CO_B);

BHELP (HE_B);

BTC (TC_B);

BCS (CS_B);

a_ACH (ACH_B);

BCOPE on BTC (b1_betw);

BHELP on BTC (b2_betw);

BCOPE on BCS (b3_betw);

BHELP on BCS (b4_betw);

a_ACH ON BTC (b5_betw);

a_ACH ON BCS (b6_betw);

model constraint:

new(betac1); !COPE on TC

new(betac2); !HELP on TC

new(betac3); !COPE on CS

new(betac4); !HELP on CS

new(betac5); !ACH on TC

new(betac6); !ACH on CS

betac1 = b1_betw - b1_with;

betac2 = b2_betw - b2_with;

betac3 = b3_betw - b3_with;

betac4 = b4_betw - b4_with;

betac5 = b5_betw - b5_with;

betac6 = b6_betw - b6_with;

new(zbetac1);

new(zbetac2);

new(zbetac3);

new(zbetac4);

new(zbetac5);

new(zbetac6);

zbetac1 = betac1*(2*sqrt(TC_B)/sqrt(CO_B));

zbetac2 = betac2*(2*sqrt(TC_B)/sqrt(HE_B));

zbetac3 = betac3*(2*sqrt(CS_B)/sqrt(CO_B));

zbetac4 = betac4*(2*sqrt(CS_B)/sqrt(HE_B));

zbetac5 = betac5*(2*sqrt(TC_B)/sqrt(ACH_B));

zbetac6 = betac6*(2*sqrt(CS_B)/sqrt(ACH_B));

output: sampstat tech4 stdyx modindices (10);
